# Modified Harada-Mori and simple wet mount to determine hookworm infections in Yo Island urban area, Songkhla, Southern Thailand

**DOI:** 10.1186/s41182-019-0156-7

**Published:** 2019-04-24

**Authors:** Sirima Kitvatanachai, Aree Taylor, Pochong Rhongbutsri, Walter R. J. Taylor

**Affiliations:** 1 Faculty of Medical Technology, Rangsit University, Lak Hok, Pathumthani 12000 Thailand; 2 Department of Preclinical Science, Faculty of Medicine, Thammasat University, Lak Hok, Pathumthani 12120 Thailand; 3 Mahidol Oxford Tropical Medicine Research Unit, Bangkok, Thailand; 4 Centre for Tropical Medicine and Global Health, University of Oxford, Oxford, UK

**Keywords:** Modified Harada-Mori, Simple wet mount, Yo island, Hookworm infections

## Abstract

**Background:**

Hookworm was a previously dominant parasitic infection in Southern Thailand. The changing population to an aging society in Yo Island has never been investigated for intestinal parasites. This study aimed to estimate the prevalence of hookworm and intestinal parasitic infections on Yo Island, a small island in Songkhla Province of southern Thailand.

**Methods:**

A cross-sectional study was conducted among volunteers aged 15 and above to give one stool sample that was screened by wet mount for intestinal parasites and the modified Harada-Mori culture (mHMFPC) which is adapted from HMFPC, using local plastic bag containers instead of test tubes for hookworm detection.

**Results:**

Two hundred forty-seven volunteers (females = 160) gave one stool. The highest participation was in age group higher than 60 years. Most were Buddhism (89.1%), agriculturist (71.4%), non-education (87.9%), and income lower than 9000 baht (50.2%). The prevalence of intestinal parasites was 13/247 (5.3%) of which 6/247 (2.4%) were positive for hookworm species *Necator americanus.* One volunteer was coinfected with hookworm and *Strongyloides stercoralis* and another with *Endolimax nana* and *Blastocystis hominis*. The mHMFPC detected more positive stool samples than wet mount and wet mount: 5 vs. 2.

**Conclusions:**

Parasite prevalence was low in this urban community of mostly low-income village dwellers. The mHMFPC appeared better at detecting hookworm but numbers were small. Combined techniques are suitable for field use.

## Background

Hookworm is a neglected tropical disease (NTDs) that has a global distribution [1, 2]. Overall, its prevalence tends to be more common in adults [3, 4]. However, soil-transmitted helminth control programs in endemic countries focus on risk group of school-aged children (2–14 years). Hookworm is found at the highest intensities in adults, and hence, its abundance is not affected by treating only school-aged children [3, 4]. The adult hookworms attach to the small intestine where they feed on blood and can cause iron-deficient anemia in individuals of all ages [5].

Over the last several decades, hookworm infection has and continues to be a significant public health problem in Thailand where studies have reported a prevalence of up to nearly 33% in schoolchildren [6–8]. One study in Nakhon Si Thammarat, southern Thailand, the mean hookworm infection rate in several primary school pupils aged 5 to < 12 years was 32.7% [9]. This contrasted to a low rate of 8% in 324 healthy volunteers from the same province [10] and 15.8% in 2014 in Southern Thailand [8].

The diagnosis of hookworm rests on finding eggs in stool samples but, currently, there is no agreement regarding which test or combination of tests should be used as the “gold standard” [11, 12].

Direct wet mount of stool has a low sensitivity for detecting light helminthic infections [13]. In two studies, wet mount had detection rates of 18.8% vs. 24.7% for formalin-ether concentration (FEC) [14], and 48.9% vs. 63.1 and 93.7% for FEC and Kato Katz tests, respectively [15]. Nuchprayoon et al. (2009) suggest that the simple direct smear is insufficiently sensitive to be used alone for stool parasite screening [16].

The sensitivity for detecting hookworm and other helminths is generally higher using the Harada-Mori filter paper culture (HMFPC). It achieved a detection rate for hookworm of 45.8% vs. 41.7% by FEC and 25.0% by simple smear in Burmese migrant workers in Thailand [16]. HMFPC also has the advantage of being able to detect third stage larvae of hookworm and *Strongyloides stercoralis* from culture and at this stage the species of hookworm can be identified [17]. The modified (m) HMFPC, using local plastic bag containers to replace the test tube which is cheap (< 0.03 USD), easy, and simple for field culture and reported higher prevalence of 11.3% compared to direct smear (6.9%) and FEC (10.1%) [18].

There are no data on the prevalence of intestinal parasites on Yo Island in southern Thailand. Therefore, we conducted a prevalence survey of hookworm and other intestinal parasites using the mHMFPC method and wet smear to inform the parasitic disease control program.

## Materials and methods

This was a cross-sectional community survey that was conducted from August to October, 2015 in Yo Island, it is a small island in Songkhla Lake in southern Thailand, approximately 15 km from Muang Songkhla that is connected to the Thai mainland via the bridge. The majority of the 4459 population live in 9 villages and make a living from agriculture, fishing, and running small businesses, e.g., hand-woven fabric. The island has two health centers [19].

The inclusion criteria were male or females aged ≥ 15 years, living in 9 villages on the island, who had voluntarily provided informed assent, had written informed consent, and provided enough stool for examination. Participants aged < 15 years living outside the area and providing an insufficient amount of stool were excluded. The sample size was calculated using single population proportion formula; assuming expected prevalence of 16% [8], 95% confidence level, 5% margin of error. The sample size was finally calculated to be 206.

Health workers distributed labeled, clean plastic containers to enrolled villagers and instructed them on how to collect the stool samples. One stool sample was collected from each participant. A structured questionnaire was used to collect data on demographics, occupation, sanitation, and hygiene practices.

Wet mount preparations were prepared in accordance with standard protocols [20]. One to two milligrams of stool were put on a slide, 0.85% NSS and 1% iodine were used for wet mount. The mHMFPC was modified from Harada and Mori [21]. Briefly, 2 g of fresh stool were placed on a folded strip of filter paper (30 mm × 150 mm), which was then placed in a 30 mm × 200 mm plastic tube containing 5 mL of sterile distilled water and incubated at room temperature (25–35 °C) for 7 days. 0.5 mL formaldehyde was then added and the tube was centrifuged at 2000 r/min for 5 min. The sediment was examined under the microscope. All third stage larvae were identified by species level based on their morphological characteristics [18, 22].

All samples were independently examined in a blinded fashion by two microscopists. Expert parasitologists reread all positive slides and 10% of randomly selected negative slides.

Data were analyzed descriptively (frequencies and percentage) using IBM SPSS Version 21.0. The prevalence of hookworm infections was determined by taking combined results of wet smear and mHMFPC. Every individual with at least one positive test was considered as truly infected.

## Results and discussion

A total of 247 villagers were able to provide stool samples for both identification methods. Most were Thai nationals, age > 50 years (> 55% of samples) and the female to male ratio was 1.9:1 (Table 1). The vast majority were Buddhist with no formal education; only 2% had a basic level of schooling. Just over 70% were agriculturist (gardeners or fishermen) while the rest had diverse occupations. Half the participants were on a low income, earning < 9000 Baht (USS 270)/month (Table 1). The characteristics of the six hookworm-positive individuals are summarized in Tables 1 and 2. They all had and used toilets, and all wore shoes when going out, usually sandals (*n* = 4); 5 of them reported that they had never been dewormed (Table 2).

**Table 1:** Socio-demographic characteristics of the 247 participants and hookworm infections in Yo Island

Demographic characteristics	No. (%)	No. of HW infections (%)
Gender		
Male	84 (34.0)	3 (3.6)
Female	160 (64.8)	3 (1.9)
No information	3 (1.2)	
Age groups (years)		
15–20	5 (2.0)	1(20)
21–30	16 (6.5)	
31–40	30 (12.2)	1(3.3)
41–50	46 (18.6)	
50–60	67 (27.1)	
> 60	70 (28.3)	4 (5.7)
No answer	13 (5.3)	
Religion		
Buddhism	220 (89.1)	6 (2.7)
Islam	2 (0.8)	
No answer	25 (10.1)	
Education		
No education	217 (87.9)	5 (2.3)
Primary and secondary school	5 (2.0)	1 (20.0)
No answer	25 (10.1)	
Occupation		
Agriculturist	105 (71.4)	2 (1.9)
Housewife	15 (6.07)	1 (6.7)
Employee	30 (12.2)	2 (6.7)
Government servant/retired	9 (3.6)	
Hairdresser	1 (0.4)	
Student	7 (2.8)	1 (14.3)
Engineer	1 (0.4)	
Private business	3 (1.2)	
Shop keeper	21 (8.5)	
Weaver	9 (3.6)	
No answer	46 (18.6)	
Income (Baht)		
< 9000	124 (50.2)	4 (3.2)
9001–16,000	30 (12.2)	1 (3.3)
16,001–30,000	16 (6.5)	
> 30,000	5 (2.0)	
No answer	72 (29.1)	1 (1.4)

**Table 2:** Sanitation and hygiene practice of the 247 participants and hookworm infections

Sanitation and hygiene practice	No. (%)	No. of HW infections (%)
Sources of drinking water		
Rain drinking	5 (2.0)	1 (20.0)
Tap water	70 (28.3)	1 (1.4)
Bottled water	121 (49.0)	3 (2.5)
Natural sources	15 (6.1)	1 (6.7)
No answer	36 (14.6)	
Toilet		
Have	239 (2.0)	6 (2.5)
No answer	8 (14.6)	
Using toilet when at work		
Yes	236 (95.6)	6 (2.5)
No	2 (0.8)	
No answer	9 (3.6)	
Wear shoes		
Yes	235 (95.1)	6 (2.6)
No	2 (0.8)	
No answer	10 (4.1)	
Type of shoes when going to work		
Sandal	141 (57.1)	6 (4.3)
General shoes	44 (17.8)	
Boots	26 (10.5)	
No answer	36 (14.6)	
Wash hand before eating and drinking		
No	3 (1.2)	
Sometimes	95 (38.5)	4 (4.2)
Always	125 (50.6)	2 (1.6)
No answer	24 (9.7)	

Combining both methods, the total number of villagers with positive stool samples was 13 for a prevalence of 5.3% (Table 3). Of these, 6 (2.4%) were infected with hookworm, including one mixed infection with *Strongyloides stercoralis*. Our study has shown that in Yo Island, that has an aging population and this is why we focused our sample on volunteers aged 15 and above. The prevalence of hookworm infection and other parasitic infections was low, just under 2.5 and 5.5%, respectively. Our data contrast with other studies from Thailand. One subnational random survey in districts from 14 provinces in southern Thailand reported an overall hookworm prevalence of 15.8% by FEC [5] while in 2002 primary school children from Nakhon Si Thammarat Province had a hookworm prevalence of ~ 20% by the Kato-Katz technique [8]. Hookworm infection in villagers from three districts in Krabi Province was 15.1% combining simple smear, mHMFPC, and mFEC in 2012 [17].

**Table 3:** The prevalence of intestinal helminths and hookworm in 247 Yo Island participants

Intestinal parasites	No. of infected (%)
*Giardia lamblia*	1 (0.4)
*Blastocystis hominis*	1 (0.4)
*Endolimax nana*	3 (1.2)
*Strongyloides stercoralis*	1 (0.4)
Hookworm	5 (2.0)
Mixed infection	
*Strongyloides stercoralis* and hookworm	1 (0.4)
*E. nana* and *B. hominis*	1 (0.4)
Total	13 (5.3)

Ours was the first intestinal parasite survey in Yo Island, which has seen increased development and urbanization over recent years. Better quality infrastructure, higher living standards with improved sanitation and personal hygiene, increasing age and a good health promotion infrastructure probably account for the low prevalence of hookworm and other parasites; such low rates are usually associated with urban environments in Thailand [23].

Only soil-transmitted helminths, hookworm, and *Strongyloides stercoralis* were found in this area. No foodborne helminths were detected, and this is probably due to good eating practice and the avoidance of raw food; indeed no one reported eating raw food (the information by health center staff).

We used the simple wet mount and the mHMFPC to determine the prevalence of intestinal parasite and hookworm infection. All positive wet mount stools were also positive by mHMFPC. The latter detected 5 cases of hookworm vs. 2 for wet mount (Table 4). The direct wet mount is useful for the observing motile protozoan trophozoites but is not recommended as the sole diagnostic test for routine stool examination because of its low sensitivity [13–15]. Nevertheless, it is easy to perform, cheap and is time-saving. In field studies, mHMFPC is a low-cost technique [< 3 Baht (USD 0.09)/test], easy to perform, and has a high sensitivity (75.3–91.6%) for detecting hookworm [16, 24]. In our study, mHMFPC detected more hookworm than wet mount (2 vs 0.8%) and combining both techniques increased detection rates. Therefore, we believe that both techniques should be used in field studies.

**Table 4:** Prevalence of hookworm and *Strongyloides stercoralis* by simple wet mount and mHMFPC

Parasites	Wet mount	mHMFPC	Total
Hookworm	2 (0.8)	5 (2.0)	6 (2.4)
*Strongyloides stercoralis*	1 (0.4)	1 (0.4)	2 (0.8)

All six cases of hookworm infection were *Necator americanus*. The identified hookworm larvae were those of *N. americanus* (100%), which is the dominant species in SE Asia [17]. Anantaphruti et al. showed 99.9% of hookworms were *N. americanus* vs. 0.1% for *A. duodenale* in southern Thailand [9]*.* Of the two, *N. americanus* is less pathogenic, with 0.02 ml of blood loss per day compared to 0.1 mL for *A. duodenale* [25].

## Conclusions

We demonstrated a low prevalence of hookworm and other parasites in a Thai population living on an island experiencing rapid economic development, suggesting good control of soil-transmitted helminths. Sensitivity was improved by the two simple and cheap techniques. Additional parasitic prevalence studies across a wider age spectrum to identify high-risk groups and tailor appropriate control efforts.
